# Correlates of total physical activity among middle-aged and elderly women

**DOI:** 10.1186/1479-5868-4-16

**Published:** 2007-05-11

**Authors:** Nicola Orsini, Rino Bellocco, Matteo Bottai, Marcello Pagano, Alicja Wolk

**Affiliations:** 1 Division of Nutritional Epidemiology, Institute of Environmental Medicine, Karolinska Institutet, Sweden; 2 Department of Medical Epidemiology and Biostatistics, Karolinska Institutet, Sweden; 3 Department of Epidemiology and Biostatistics, Arnold School of Public Health, University of South Carolina, USA; 4 Department of Biostatistics, Harvard School of Public Health, USA; 5 Department of Statistics, University of Milano Bicocca, Italy

## Abstract

Information on correlates of total physical activity (PA) levels among middle-aged and elderly women is limited. This article aims to investigate whether total daily PA levels are associated with age, body mass index, smoking, drinking status, and sociodemographic factors.

In a cross-sectional study of 38,988 women between the ages of 48 and 83 years residing in central Sweden, information on PA, weight, height, smoking, drinking, and sociodemographic factors was collected through a self-administered questionnaire. Total daily PA levels were measured as metabolic equivalents (MET-h/day). Odds ratios (OR) and 95% confidence intervals (CI) were estimated by ordinal logistic regression models.

We observed decreasing level of total PA with increasing age (for 5-year increase: OR = 0.87; 95% CI: 0.85–0.89) and body mass index (for 5-unit, kg/m^2^, increase: OR = 0.81; 95% CI: 0.79–0.84). Multivariable adjusted correlates of total PA level were smoking (current vs. never: OR = 0.83; 95% CI: 0.79–0.88), drinking (current vs. never: OR = 0.88; 95% CI: 0.82–0.94), educational level (university vs. primary: OR = 0.54; 95% CI: 0.51–0.58), employment status (housewife vs. full-work: OR = 2.59; 95% CI: 2.25–2.98), and childhood environment (city vs. countryside: OR = 0.62; 95% CI: 0.59–0.65).

In the present investigation, among middle-aged and elderly women, the likelihood of engaging in higher total daily PA levels decreased with age, body mass index, educational level, smoking, drinking, and growing up in urban places.

## Background

An extensive body of epidemiological evidence has shown positive associations between regular physical activity (PA) and health benefits [1]. Several studies support the important role of PA for both primary and secondary prevention of cardiovascular diseases [2]. There is also evidence that regular PA may prevent osteoporosis [3], some forms of cancer [4], type 2 diabetes [5,6], and may increase longevity [7].

According to a review of correlates of participation in physical activity, middle-and older age groups are more likely to engage in low levels of physical activity than younger age groups, and women are more likely to be inactive than men [8]. In addition, many of the studies included in this review did not incorporate different domains of PA and focused mainly on leisure-time PA [8]. To our knowledge there are no population-based studies investigating different types of correlates of total PA levels among middle-aged and elderly women.

The aim of this study was to investigate, in a cross-sectional setting, the association between age, body mass index, smoking and drinking status, and sociodemographic factors and total daily PA levels in a large population-based cohort of Swedish women.

### Study population

The Swedish Mammography Cohort (SMC87) was established between 1987 and 1990, when all (90,303) women aged 40–75 years and living in Västmanland and Uppsala County received a mailed questionnaire about diet, weight, height, and education. Completed questionnaires were obtained from 66,651 women in the source population (74%). We excluded women with missing or incorrect identification codes; missing date of the questionnaire; and missing date of death. After these restrictions our baseline cohort consisted of 61,433 women. In 1997 a more detailed questionnaire (SMC97) was sent to the members of the cohort. This questionnaire included a detailed food frequency questionnaire as well as questions on weight, height, smoking status, alcohol intake, educational level, childhood environment, employment status, and PA. The questionnaire was sent to 56,054 women who participated in the first wave, were alive, and had not moved out from the study area. The response rate was 70% in the SMC97. The study was approved by the Ethics Committees at the Uppsala University and the Karolinska Institutet. Obtaining written information about the study and completion of the questionnaire were considered to imply informed consent.

### Physical activity assessment

Measurement of PA was based on a self-administered questionnaire. Five types of past year physical activities were estimated: home/household work, walking/cycling, work/occupation activity, TV/reading, and exercise. To calculate the activity score of specific type of activity, the intensity of these activities defined as metabolic equivalents (MET, kcal/kg/hour) was multiplied by reported time (hours) [5]. An open question asked about time spent sleeping, out of 24 hours per day. We estimated a total daily PA score by adding up the specific activities together [9]. We corrected the self-reported time to 24 hours per day, by adding hours (if the total sum was below 24 hours) or deleting hours (if the total was above 24 hours). This correction time was multiplied by the intensity factor of 2 MET, corresponding to the mean of self-care/walking at home (2.5 MET) and sitting (1.5 MET). This correction was based on the assumption that underestimation of time might be due to these common activities not being asked for in the questionnaire [10]. The PA questionnaire was validated against 2×7-days of activity diary in a group of Swedish men between the ages of 44 to 78 years and was shown to estimate total physical activity satisfactorily (Spearman correlation between the questionnaire and PA records was 0.6) [11]. Reproducibility of the PA questionnaire was evaluated in a subset of the SMC97 and it was shown to be acceptable (Intraclass correlation was 0.7 for the total activity score)[12].

### Statistical analysis

To make description and interpretation of the associations easier, we categorized the total daily PA score into quintiles. We used an ordinal logistic regression model, also known as proportional odds model or cumulative logit model, to estimate associations (odds ratios and 95% confidence intervals) between levels of total PA and each correlate in univariable and multivariable models [13]. In this model the estimated odds ratio does not depend on the PA quintiles being compared (≥ *k *vs. <*k*, where *k *ranges between the second to the fifth quintile of PA).

The correlates included in the final model were age in groups (48–54; 55–59; 60–64; 65–69; 70–74; 75–79; 80–83 years); body mass index (BMI <25, 25–29, and ≥ 30 kg/m^2^); alcohol intake (never, former, and current); smoking status (never, former, and current); postmenopausal status; education (primary school, high, and university); employment status (full-work, part-time, housewife, retired, pension disability, and unemployed); and childhood environment (country side, small town, and city). We used a Wald test to assess whether each correlate (one or more non-referent odds ratios) was statistically significant at 5% level. All statistical analyses were performed using the statistical package Stata (StataCorp LP, Texas, USA).

## Results

Our study cohort consisted of 38,988 women between the ages of 48 and 83 years. The mean age was 62 ± 9 years, and mean BMI was 25 ± 4 kg/m^2^. The mean value of total daily PA was 42.2 ± 4.8 MET-h/day. On average, the major contributors to the 24 hours total activity score were occupational (12.1 MET-h/day), household (8.6 MET-h/day) activities and sleeping (6.4 MET-h/day), then, in order, leisure time physical inactivity (2.9 MET-h/day), walking/bicycling (2.3 MET-h/day) and exercise/sports activities (1.7 MET-h/day).

Cross-tabulations between each correlate and PA levels, together with multivariable adjusted associations are shown in Table 1. We observed a decreasing linear association between total PA and age (for 5-year increase: odds ratio (OR) = 0.87; 95% confidence interval (CI): 0.85–0.89) and BMI (for 5-unit, kg/m^2^, increase: OR = 0.81; 95% CI: 0.79–0.84). The odds of having higher PA level decreased by 40% (95% CI: 0.56–0.64) for obese women (BMI ≥ 30) when compared with normal weight women. Postmenopausal women were slightly more likely to engage in higher PA levels (OR = 1.09; 95% CI: 1.02–1.15). Being a current drinker or smoker was associated with a decreased likelihood of being classified into the highest category of PA levels. The odds of having higher PA levels decreased by 17% (95% CI: 0.79–0.88) for current smokers when compared with women who never smoked. Higher levels of education (university) were associated with a statistically significant decreased likelihood of having higher PA levels (OR = 0.54; 95% CI: 0.51–0.58). We found marked differences in the total PA levels by employment status. The odds of having higher PA levels was considerably greater among part-time workers (OR = 1.96; 95% CI, 1.85–2.09) and housewives (OR = 2.59; 95% CI: 2.25–2.98) as compared to full-time workers. Growing up in the city, as compared to the countryside, was associated with 38% decrease in the likelihood of engaging in higher levels of PA (95% CI, 0.59–0.65).

**Table 1:** Frequency counts and multivariable odds ratios with 95% confidence intervals for each correlate of total physical activity levels.

	**Total physical activity, quintiles**						
			
	**'Very Low'**	**'Low'**	**'Moderate'**	**'High'**	**'Very High'**		
**Median MET-h/day**	**36**	**39**	**42**	**45**	**49**	**Total **^2^	
			
**Characteristics**	**No. (%)**	**No. (%)**	**No. (%)**	**No. (%)**	**No. (%)**	**No. (%)**	**OR (95% CI) **^3^
**Age group, years**							
48–54	2158(21)	2243(22)	2046(20)	1940(19)	1835(18)	10,222(32)	1.00 ^1 ^*
55–59	1141(19)	1194(20)	1218(20)	1207(20)	1287(21)	6047(19)	1.04 (0.98, 1.11)
60–64	883(19)	848(18)	919(20)	1012(21)	1045(22)	4707(15)	0.98 (0.90–1.06)
65–69	659(17)	742(19)	804(20)	857(22)	883(22)	3945(13)	0.85 (0.75–0.97)
70–74	649(19)	656(20)	659(20)	675(20)	686(21)	3325(11)	0.68 (0.59–0.77)
75–79	593(23)	500(20)	512(20)	478(19)	447(18)	2530(8)	0.54 (0.47–0.62)
80–83	202(32)	105(16)	121(19)	114(18)	94(15)	636(2)	0.38 (0.31–0.46)
**Body mass index, kg/m**^2^							
< 25	3168(18)	3605(21)	3595(20)	3594(20)	3524(19)	17,486(56)	1.00 ^1 ^*
25–29	2138(22)	1984(19)	2014(19)	2098(20)	2096(20)	10,330(33)	0.86 (0.82–0.90)
≥ 30	895(29)	632(20)	597(18)	517(15)	580(18)	3221(11)	0.60 (0.56–0.64)
**Postmenopausal status**							
No	1390(21)	1493(23)	1380(21)	1257(19)	1086(16)	6606(23)	1.00 ^1 ^*
Yes	4474(20)	4367(19)	4451(20)	4577(20)	4652(21)	22,521(77)	1.09 (1.02–1.15)
**Drinking status**							
Never	636(18)	610(16)	712(20)	736(21)	880(25)	3574(12)	1.00 ^1 ^*
Former	601(21)	481(17)	543(19)	577(20)	641(23)	2843(9)	0.95 (0.86–1.05)
Current	5018(20)	5150(21)	4980(20)	4926(20)	4685(19)	24,759(79)	0.88 (0.82–0.94)
**Smoking status**							
Never	2954(18)	3174(19)	3307(20)	3407(22)	3443(21)	16,285(52)	1.00 ^1 ^*
Former	1867(22)	1836(21)	1749(20)	1607(19)	1548(18)	8607(28)	0.88 (0.84–0.93)
Current	1393(23)	1209(20)	1129(19)	1168(19)	1180(19)	6079(20)	0.83 (0.79–0.88)
**Education**							
Primary School	2495(17)	2484(17)	2830(19)	3251(22)	3815(25)	14,875(48)	1.00 ^1 ^*
High School	2180(22)	2034(21)	1897(20)	1813(19)	1759(18)	9683(31)	0.68 (0.64–0.71)
University	1565(24)	1723(26)	1513(23)	1170(18)	637(9)	6608(21)	0.54 (0.51–0.58)
**Employment status**							
Full-work	2432(23)	2555(24)	2113(20)	1794(18)	1592(15)	10,486(34)	1.00 ^1 ^*
Part-time	680(12)	854(15)	1161(20)	1420(25)	1567(28)	5682(18)	1.96 (1.85–2.09)
Housewife	97(10)	102(11)	183(20)	226(25)	320(34)	928(3)	2.59 (2.25–2.98)
Retired	2174(20)	2099(19)	2177(20)	2232(21)	2185(20)	10,867(35)	1.35 (1.21–1.51)
Pension disability	651(29)	420(19)	436(20)	363(16)	365(16)	2235(7)	0.75 (0.68–0.82)
Unemployed	222(20)	232(21)	194(18)	225(21)	214(20)	1087(3)	1.11 (0.98–1.25)
**Grow-up**							
Countryside	2540(16)	2670(17)	2910(19)	3446(22)	3858(26)	15,424(49)	1.00^1 ^*
Small town	1664(22)	1703(22)	1637(21)	1392(18)	1254(17)	7650(25)	0.70 (0.66–0.74)
City	2018(25)	1852(23)	1667(21)	1366(17)	1087(14)	7990(26)	0.62 (0.59–0.65)

^1 ^Referent group.

^2 ^Univariate frequency counts of the covariate with column percentage within parenthesis.

^3 ^OR, odds ratio; CI, confidence interval estimated with a multivariable ordinal logistic regression model on complete cases (*n *= 27,789). The effects of the covariates are mutually adjusted.

* p-value < 0.05 of a Wald test. The null hypothesis is that the non-referent odds ratios are equal to 1.

## Discussion

In this analysis of a cohort of middle-aged and elderly women in Sweden we found that the likelihood of engaging in high levels of total PA decreased with age, body mass index, and educational level. Behavioral factors such as drinking and smoking were inversely associated with total PA level. Furthermore, women who grew up in the city were less likely to be in the highest category of total PA levels.

This study had many strengths. Our large population-based cohort of 38,988 women is representative of the Swedish female population in regard to age range, educational status and relative weight [14]. The quantitatively estimated total daily PA is based on a self-administered questionnaire with acceptable validity and reproducibility [11,12].

One of the concerns of this analysis is that women may not accurately recall their activities. The misclassification in the total activity score is likely to be nonsystematic and would attenuate any associations between total PA and correlates. Although self-reported PA has its limitations, in large population-based cohort studies it is more feasible than the gold standard (doubly labeled water) [15].

One of the difficulties when comparing our study with others is the variation in PA questionnaires and types of measurements used. Quantifying the level of leisure time exercise only may give a distorted understanding of total PA in a population. This is especially true for women who are more likely to engage in light or moderate activities. The majority of previous studies among women examined the influence of individual, social, and environmental factors on some aspects of PA behavior (mostly leisure-time or sport/exercise activities) [8,16-18].

Our results, that PA declines with age, are in agreement with previous studies that were based on doubly labeled water [19]. BMI and PA are inextricably linked. Our results support numerous studies that found an inverse association between PA and obesity [20-22]. It is known that health-risk behaviors such as smoking, drinking, and inadequate levels of PA tend to cluster together [16], as we indeed observed in the present study. A possible explanation of the weak positive association between postmenopausal status and total PA is that postmenopausal status could act as a proxy for unmeasured factors associated with total PA. For instance, postmenopausal women are more likely to be retired and therefore have more free time available (lack of time constraints).

In our study occupational and household activities were two major contributors of the total daily activity score that is a combination of intensity and duration. Educational level was inversely associated with total daily PA level perhaps because higher educated women tend to have lighter or more sedentary jobs and less time for household activities [23]. Previous studies that focused on leisure-time PA only found that higher levels of education were associated with higher levels of leisure-time PA [24,25]. In addition, a study among Australian women (mean age 43 years) also found the positive association between higher occupational status and leisure-time PA, which remained unchanged even after taking into account occupational/home PA [26].

A comparison of different studies, however, should be based on the same type of physical activities and age range. Estimating only leisure-time activities may however give an unclear picture of the levels of total PA, since a heavy manual worker with no leisure-time activity would be classified as inactive, while a sedentary worker that engages in volleyball twice a week would be classified as active. Therefore, findings that are not entirely consistent across studies highlight the importance of assessing different domains of PA [26].

A recent review about correlates of adults' participation in PA shows how different factors (demographic, biological, psychological, emotional, social, cultural and environmental) can affect PA patterns [8]. In particular, over the past decade there has been a growing recognition of the role of the environment in affecting healthy behaviors [27]. Our finding that the environment in which these women grew up is related to the current total PA seems to support a life course approach to chronic disease epidemiology where time and timing of exposure-disease associations are important [28]. Furthermore, our findings seem to support previous studies showing higher PA levels in women residing in rural areas as compared to urban areas [29-31]. Given that PA is a complex behavior, the intensity and the direction of these associations might differ between populations. For instance, a study among US women aged 40 years and older found that rural women were less likely to engage in high leisure time activity levels in comparison to women residing in urban areas [25]. A possible explanation of these discrepancies is the different set of characteristics (socio-demographic and environment) associated with urban and rural settings in different areas of the world.

## Conclusion

Our findings contribute to evidence of the correlates of such complex behavior as physical activity among middle-aged and elderly women. Since engaging and maintaining regular PA level plays a key role in reducing several public health problems, the identification of significant correlates may help researchers, clinicians, and health policy makers to design gender-specific interventions.

In summary, in this study among middle-aged and elderly women, the likelihood of engaging in higher total daily PA levels decreased with age, body mass index, educational level, smoking, drinking, and growing up in urban places.

## Competing interests

The author(s) declare that they have no competing interests.

## Authors' contributions

NO analyzed the data and drafted the manuscript incorporating critical inputs from all authors. AW is a principal investigator of the cohort; she conceived the study, participated in its design and coordination. RB, MB, MP, and AW provided critical revision of the paper and assisted with the analysis and interpretation. All authors have read and approved the final manuscript.
